# Identification of Early-Onset Metastasis in SF3B1 Mutated Uveal Melanoma

**DOI:** 10.3390/cancers14030846

**Published:** 2022-02-08

**Authors:** Wojtek Drabarek, Job van Riet, Josephine Q. N. Nguyen, Kyra N. Smit, Natasha M. van Poppelen, Rick Jansen, Eva Medico-Salsench, Jolanda Vaarwater, Frank J. Magielsen, Tom Brands, Bert Eussen, Thierry. P. P. van den Bosch, Robert M. Verdijk, Nicole C. Naus, Dion Paridaens, Annelies de Klein, Erwin Brosens, Harmen J. G. van de Werken, Emine Kilic, on behalf of the Rotterdam Ocular Melanoma Study Group

**Affiliations:** 1 Department of Ophthalmology, Erasmus MC Cancer Institute, Erasmus MC, 3000 CA Rotterdam, The Netherlands; w.drabarek@erasmusmc.nl (W.D.); j.nguyen@erasmusmc.nl (J.Q.N.N.); kyra.smit@gmail.com (K.N.S.); n.vanpoppelen@erasmusmc.nl (N.M.v.P.); j.vaarwater@erasmusmc.nl (J.V.); t.brands@erasmusmc.nl (T.B.); n.naus@erasmusmc.nl (N.C.N.); D.Paridaens@oogziekenhuis.nl (D.P.); 2 Department of Clinical Genetics, Erasmus MC Cancer Institute, Erasmus MC, 3000 CA Rotterdam, The Netherlands; e.medicosalsench@erasmusmc.nl (E.M.-S.); f.magielsen@erasmusmc.nl (F.J.M.); h.eussen@erasmusmc.nl (B.E.); a.deklein@erasmusmc.nl (A.d.K.); e.brosens@erasmusmc.nl (E.B.); 3 Department of Medical Oncology, Erasmus MC Cancer Institute, Erasmus MC, 3000 CA Rotterdam, The Netherlands; j.vanriet@erasmusmc.nl; 4 Cancer Computational Biology Center, Erasmus MC Cancer Institute, University Medical Center, 3000 CA, Rotterdam, The Netherlands; rick.jansen1984@gmail.com; 5 Department of Urology, Erasmus MC Cancer Institute, University Medical Center, 3000 CA Rotterdam, The Netherlands; 6 Department of Pathology, Section Ophthalmic Pathology, Erasmus MC Cancer Institute, Erasmus University Medical Center, 3000 CA Rotterdam, The Netherlands; t.vandenbosch@erasmusmc.nl (T.P.P.v.d.B.); r.verdijk@erasmusmc.nl (R.M.V.); 7 The Rotterdam Eye Hospital, 3011 BH Rotterdam, The Netherlands; 8 Department of Immunology, Erasmus MC Cancer Institute, University Medical Center, 3000 CA Rotterdam, The Netherlands

**Keywords:** uveal melanoma, *SF3B1* mutation, aberrant splicing, early metastasis, RNA-seq

## Abstract

**Simple Summary:**

This study describes clinical and genetic characteristics of the largest aggregated cohort of Splicing Factor 3 Subunit B1 (*SF3B1*)-mutated Uveal Melanoma (UM) in the literature (*n* = 146). Missense mutations in the spliceosome gene *SF3B1* result in an altered splice site recognition and aberrant mRNA transcripts. The *SF3B1*-mutated UM show early- and late-onset of metastatic disease for which, currently, no distinguishing biomarkers exist. Using a cutoff of 60 months for stratification, we found that a largest basal tumor diameter was more prevalent in the early-onset metastatic disease group. Furthermore, using differential gene expression and the detection of aberrant transcripts, we found that the expression of alpha/beta-Hydrolase domain containing 6 (*ABHD6)* is associated with early-onset metastatic *SF3B1* and aberrant transcripts that are associated with early-onset *SF3B1*-mutated UM. Our results provide more accurate prognostication and targets for future functional studies in an effort to elucidate pathogenesis of *SF3B1*-mutated UM.

**Abstract:**

Approximately 25% of all uveal melanoma (UM) contain driver mutations in the gene encoding the spliceosome factor *SF3B1*, and whilst patients with such *SF3B1* mutations generally have an intermediate risk on developing metastatic disease, a third of these patients develop early metastasis within 5 years after diagnosis. We therefore investigated whether clinical and/or genetic variables could be indicative of short progression-free survival (PFS < 60 months) or long PFS (PFS ≥ 60 months) for *SF3B1*-mutated (*SF3B1*^mut^) UM patients. We collected 146 *SF3B1*^mut^ UM from our Rotterdam Ocular Melanoma Studygroup (ROMS) database and external published datasets. After stratification of all *SF3B1*^mut^ UM using short PFS vs. long PFS, only largest tumor diameter (LTD) was significantly larger (mean: 17.7 mm (±2.8 SD) in the short PFS *SF3B1*^mut^ group vs. the long PFS group (mean: 14.7 (±3.7 SD, *p* = 0.001). Combined ROMS and The Cancer Genome Atlas (TCGA) transcriptomic data were evaluated, and we identified *SF3B1*^mut^-specific canonical transcripts (e.g., a low expression of *ABHD6* indicative for early-onset metastatic disease) or distinct expression of *SF3B1*^mut^ UM aberrant transcripts, indicative of early- or late-onset or no metastatic *SF3B1*^mut^ UM.

## 1. Introduction

Uveal melanoma (UM) is a highly malignant tumor with metastatic capacity. Metastatic disease is detected either early (<60 months) or late during follow-up. Staging of primary UM has been performed using American Joint Committee on Cancer (AJCC) [1] criteria, but studies show that prognostication of UM patients is also possible through analysis of chromosomal rearrangements [2], sequencing of UM driver genes [3,4], and evaluating gene expression profiles (GEP) [5]. Adding chromosome 3 and 8q status to AJCC classification improves accuracy of prognostication of UM patients [6]. Gain-of-function mutations in guanine nucleotide-binding protein subunit alpha (Gαq) *(GNAQ*), guanine nucleotide-binding protein alpha 11 (*GNA11*) (or, more rarely, in cysteinyl leukotriene receptor 1 *(CYSLTR2*) or phospholipase C beta 4 *(PLCBC4*)) are considered primary driver events which are found in almost all UM but are not associated with patient prognosis. Mutations in secondary UM driver genes are strongly associated with prognosis of UM patients and affect BRCA1-associated protein 1 (*BAP1;* associated with the worst prognosis), splicing factor 3b subunit 1 (*SF3B1;* associated with intermediate prognosis), and eukaryotic translation initiation factor 1A X-Linked (*EIF1AX;* associated with the most favorable prognosis). BAP1 is an enzyme involved in deubiquitination and interacts with different proteins such as DNA damage repair protein breast cancer type 1 (BRCA1). Splicing Factor 3b Subunit 1 (*SF3B1*) mutations occur in 15–29% of UM [7,8,9,10] and are cytogenetically characterized by multiple distal chromosomal copy number variations (CNV) such as (partial) loss of chromosome 1p and chromosome 6q and gain of chromosome 6p or chromosome 8q [11,12]. In The Cancer Genome Atlas (TCGA) milestone paper of Robertson et al., most *SF3B1*-mutated UM were allocated in cluster two comprising disomy chromosome 3 and chromosome 8q gain [12]. Moreover, this cluster analysis was superior in prognostication than the AJJC classification [13]. Finally, EIF1AX is a protein involved in stabilizing the ribosomes during translation, which is also an essential cellular process [14]. These UM tumors occur in approximately 20% of all UM and rarely metastasize.

Mutations in *SF3B1* in UM occur mostly at the gene position that encode the amino acid (AA) residues 625, and more rarely affected AA-residues are 666, 700, 783, 781, 742, and 1123 [15]. SF3B1 is involved in splicing of the precursor mRNA, which is an essential cellular process in all eukaryotic species. *SF3B1* mutations occur in a heterozygous state and are change-of-function mutations that result in a broad range of aberrantly spliced transcripts due to the mutant SF3B1 protein, as encoded by the mutant allele. The wild-type allele remains active to produce the canonical spliced transcripts. The aberrant transcripts are the result of the use of alternative recognition sites by the mutated spliceosome complex and thereby utilizing (or preferring) a non-canonical splice site due to genomic mutations encoded within the Heat Domains of *SF3B1* [16]. Somatic missense mutations predominantly affect the 625 arginine residue within one of the heat domains of SF3B1 [16]. This peculiar preference is in contrast to other malignancies such as breast cancer and leukemia, in which related *SF3B1* amino acid substitutions more frequently affect residue K700 and K666, respectively. In the TCGA-Uveal Melanoma cohort (TCGA-UVM) [12], 14 out of the 18 somatic *SF3B1* mutations detected reside within the residue R625. In the remaining four cases, *SF3B1* mutations reside twice within the residue K666 and once within T663 and H662 and therefore account for 22% of non-R625 *SF3B1* mutations.

To predict early- (PFS < 60 months) or late- (PFS ≥ 60 months) onset metastatic disease in the *SF3B1* mutated (*SF3B1*^mut^) UM, we first set out to describe clinical characteristics of *SF3B1*^mut^ UM from the updated ROMS cohort (*n* = 48) [11,17] and compare these to an aggregated cohort distilled from literature. Secondly, we utilize clinical data, whole-transcriptome datasets comprising 106 UM (26 ROMS and 80 TCGA) with mutated secondary driver genes, evaluate differentially expressed genes, and explore differentially expressed aberrantly spliced transcripts that characterize *SF3B1*^mut^ UM. Finally, we hypothesize that canonical gene expression or aberrantly spliced transcript expression can be used to discriminate between early-onset metastatic disease (defined as progression free survival (PFS) < 60 months) and late-onset metastatic disease defined as PFS ≥ 60 months *SF3B1*^mut^ UM patients.

## 2. Materials and Methods

### 2.1. Generation of a Uniform Clinical Dataset of UM Patients

**Figure 1:**
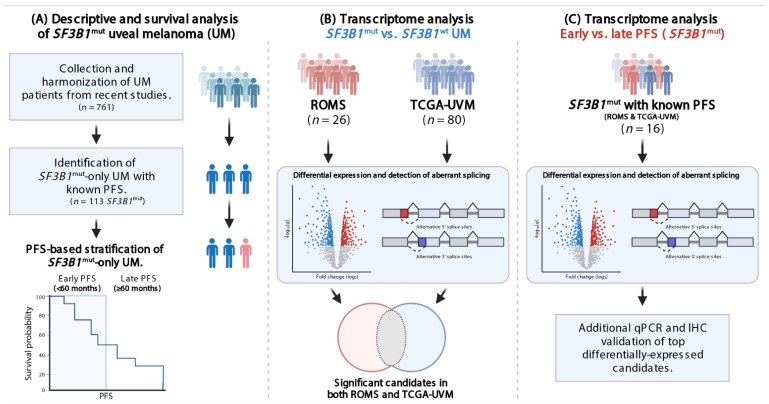
Flowchart visualizing study design and aims. (**A**) Data from *SF3B1*^mut^ UM from 11 cohorts were collected and described with stratification criteria. (**B**) Differential gene expression analysis (DGE) was performed and the ROMS, and TCGA-UVM data were intersected to investigate *SF3B1*^mut^ specific transcripts. (**C**) DGE was performed on *SF3B1*^mut^-only samples using the PFS stratification criteria to investigate transcript expression characteristic for early onset (PFS < 60) and late onset (PFS ≥ 60 months). Finally, in silico results were validated in vitro.

We combined clinical and genetic variables from 10 publicly available datasets: Alsafadi et al. [8], Royer-Bertrand et al. [18], Johnson et al. [19], Furney et al. [7], Harbour et al. [9], Shain et al. [20], Martin et al. [10], Zehir et al. [21], Rodrigues et al. [22], and Robertson et al. [12] (TCGA-UVM) cohorts. We updated the ROMS dataset used by Yavuzyigitoglu et al. [11] until 2019 and generated a dataset comprising these 11 study groups (Supplementary Figure S1). An overview of the study and study aims is depicted in Figure 1. Clinical and histopathological parameters such as age at diagnosis, gender, ciliary body involvement, presence of epithelioid cells, extraocular extension, closed extracellular matrix patterns, largest tumor diameter (LTD), tumor thickness, T class in TNM [23], inflammation, necrosis, metastatic disease, progression-free survival, patient status, *GNAQ*/*GNA11* gene mutation status, *SF3B1* mutation status, and corresponding amino acid changes and/or nucleotide changes were included. The rationale behind PFS cutoff of 60 months was based on a previously observed bimodal metastatic potential [24] and due to an approximately even distribution of samples at risk in the PFS < 60 months and PFS ≥ 60 months groups in survival analyses. Student’s *t*-test was applied for continuous variables, whereas Fisher’s Exact test was applied for categorical variables. *p*-value < 0.05 was considered statistically significant.

### 2.2. Mutation Analysis

Mutation status of *BAP1*, *SF3B1*, *GNAQ*, *GNA11*, and *EIF1AX* was determined for all UM with either BAP1 immunohistochemistry (IHC), Sanger sequencing, and/or next-generation sequencing, as previously described [4,25,26]. Additionally, we used RNA sequencing data from 26 primary UM patients with *BAP1*, *SF3B1*, or *EIF1AX* mutations, which were acquired as described previously by Smit et al. [27]. Further analyses include samples with solely a *SF3B1* mutation and for which we exclude UM with concomitant mutations in either *BAP1* or *EIF1AX* (*n =* 10).

### 2.3. Survival Analysis

Progression-free survival (PFS) was determined as the interval from treatment until metastasis or metastasis and subsequent death due to UM or until last follow-up. If the interval from treatment to metastatic disease and interval from treatment to death due to UM was reported, we used the interval from treatment to death due to UM or development of metastasis as study endpoints. Patients were censored when they were lost to follow-up or when death from other cause than UM occurred, or occurrence of death was reported without any cause.

### 2.4. Processing and Analysis of Whole-Transcriptome Data

We used whole-transcriptome sequencing data from 26 UM; information on sample acquisition, preparation, and sequencing has been described previously by Smit et al. [27]. In brief, total RNA was isolated from 5 μm sections of snap-frozen uveal melanoma samples, using the Qiagen miRNeasy isolation kit (Qiagen, Hilden, Germany) according to the manufacturer’s protocols. Consequently, transcripts longer than 200 nucleotides were sequenced on the Ion Proton sequencer (Thermofisher Scientific, Waltham, MA, USA) to produce single-end sequencing reads.

The whole-transcriptome BAM files for the TCGA-UVM cohort (*n* = 80) were downloaded from NCBI dbGaP website in 2019, under phs000178.v10.p8. Sample acquisition, library preparations and sequencing protocols of the TCGA-UVM cohort were described previously by The Cancer Genome Consortium [12].

Whole-transcriptome BAM files from the TCGA-UVM cohort were sorted on read-names (natural sort) and converted back to unmapped paired-end reads with Samtools (v1.7; htslib v1.9) [28] and Sambamba (v0.7.0) [29]. All whole-transcriptome samples (ROMS and TCGA-UVM) were mapped against the human reference genome (GRCh38; GenBank accession: GCA_000001405.15) using STAR (v2.7.1a) [30] with genomic annotations from GENCODE (v30) [31]. After alignment, duplicate reads were marked, and alignment metrics were obtained using Sambamba (v0.7.0) [29]. To identify potential alignment issues regarding transcript read uniformity, transcript integrity numbers were calculated using tin.py (v2.6.6) [32]. Read counting was performed using featureCounts (v1.6.3) [33] on the GENCODE (v30) genomic annotations; only primary-aligned reads overlapping exonic regions were counted and summarized per gene. General sequence characteristics are visualized in Supplementary Figure S2.

### 2.5. Differential Gene-Expression Analysis

Raw read counts for GENCODE (v30) transcripts were used as input for DESeq2 (v1.24.0) [34], with the exclusion of pseudogenes, mitochondrial RNA, ribosomal RNA, immunoglobulin (Ig)-variable chain, and T-cell receptor (TcR) genes and inactivated immunoglobulin genes (*n* = 41140).

For both the ROMS and TCGA-UVM cohort separately, a Wald test between *SF3B1*^mut^-only samples and *SF3B1*^wildtype (wt)^ samples (excluding any double-mutants and/or *SRSF2*^mut^ samples) was performed. An additional Wald test between *SF3B1*^mut^-only samples based on PFS < 60 months with metastatic disease and PFS ≥ 60 months was performed on the combined (ROMS and TCGA-UVM) cohort. Hereafter, PFS < 60 months refers to UM patients with early metastatic disease and late PFS (PFS ≥ 60 months) refers to patients with late or without metastatic disease. During differential gene expression analysis, we corrected for gender batch-effect in all analyses and additionally for cohort batch effect (ROMS/TCGA-UVM) in the combined cohort. To correct for multiple hypothesis testing after DESeq2 analysis, we employed independent hypothesis weighting (IHW; v1.12.0) [35]. Fold-changes (log_2_) were shrunk using their respective coefficient using apeglm (v1.6.0) [36]. A principal component analysis (PCA) was performed on the top 5000 most-variable (row-wise) genes after correcting for batch effects (Supplementary Figure S3). The top differential candidates were selected based on the following criteria: log_2_ fold change ≥ |0.5|, adjusted *p* (q) ≤ 0.05, and average read counts of ≥10 over all samples.

### 2.6. Differential Gene-Set Enrichment Analysis (GSEA)

Using the R package fgsea (v1.10.0) [37] with 100.000 permutations, we performed gene-set enrichment analysis using the Wald-statistics obtained from the prior DESeq2 analysis for all transcripts with at least 1 read on average to reduce rank ties for low-coverage transcripts. We tested the KEGG (*n* = 186) and HALLMARK (*n* = 50) gene sets (version 7.0), which contain 7732 distinct genes in all of the chosen gene sets obtained from the Molecular Signatures Database (MSigDB) [38], for statistically significant enrichment or depletion (q ≤ 0.05). Prior to testing, the ENTREZ identifiers of the gene sets were converted into ENSEMBL identifiers, of which 17 ENTREZ identifiers could not be mapped to ENSEMBL identifiers and were discarded as part of their respective gene set(s).

### 2.7. Detection of Aberrant Splicing Patterns

To detect *SF3B1*-related alternative splicing, we performed a strategy using a custom in-house workflow designed around STAR and DEXSeq [39]. This workflow was specifically designed for the detection of aberrant 5′ and 3′ exon shortenings and extensions. The workflow detected aberrant splicing events by incorporating the novel splice-events detected by STAR (SJ.out.tab) during the initial alignment procedure, as novel exonic regions within the GENCODE (v30) genomic annotations. These novel exonic regions were assigned to the nearest neighboring up- and downstream canonical exon (respective to orientation) within GENCODE (v30) annotations and assigned the respective genomic annotations of this neighboring exon, e.g., the gene name and ENSEMBL identifier, among others, and saved as a custom GFF3 file. The new custom GFF3 file contained the novel exonic portions per gene, next to the canonical annotations. We used the subread_to_DEXSeq (https://github.com/vivekbhr/Subread_to_DEXSeq accessed on 25 November 2019) script to further process our custom annotation (GFF3) and create a flattened version, in which each exon is split into its constituent non-overlapping exonic portions. Subsequently, this custom-flattened GFF3 file was used to count the reads per overlapping exonic portion using featureCounts (v1.6.3) [33]. These exon expression read counts were imported into DEXSeq (v1.30.0) [39] to detect differential 5′ and 3′ splice-sites and exons between *SF3B1*^mut^ and *SF3B1*^wt^ samples and *SF3B1*^early^ and *SF3B1*^late^ samples for both ROMS and the TCGA-UVM cohort.

To determine statistically significant differentially expressed exonic regions, we used the following criteria: adjusted *p*-value ≤ 0.05 and a log_2_ fold-change of the splicing event ≥|0.5|. In downstream analysis, we denoted the exonic regions (acceptor/donor) not-yet-present within the GENCODE v30 annotation as novel (acceptor/donor) splicing aberrations.

### 2.8. Validation of In Silico Results

Within our cohort consisting of 48 ROMS samples, there was tumor material available of 31 Formalin Fixed Paraffin Embedded (FFPE) UM to validate in silico results on with IHC. Of these samples, 20 fresh frozen UM patient samples were available for RNA isolation, as described previously by Smit et al. [27], of which 10 samples were of sufficient quality. Next, seven samples served as an independent validation set. In addition, three samples that were utilized in DGE expression analysis functioned as a validation set of RNA-seq results. Finally, several biomarkers (*ABHD6*, *CSRNP1*) derived from the aforementioned DGE analyses were validated with quantitative PCR (qPCR). Then, a 500 ng (nanogram) RNA input was used in cDNA conversions, except for one (PFS < 60 months) *SF3B1*^mut^ sample, for which a maximum input of 450 ng RNA was possible due to concentration restraints. A real-time PCR (RT-PCR) reaction mix consisted of 5- or 7-times diluted cDNA, 10 μL iTaq Universal SYBR Green Supermix (Bio-rad, Hercules, CA, USA), and 10 μM forward and reverse primers, which were placed in a CFX96 real-time system (Bio-Rad, Hercules, CA, USA). Delta cT values of genes of interest were calculated relative to the control transcript *CHMP2A*. All qPCR reactions were successfully performed in technical triplicates, except for sample 7, for which only two cT values for *CHMP2A* could be used. Differential gene expression was calculated using the threshold cycle (Ct) method [40]. A *SF3B1*^mut^ UM sample with 45.5 months PFS with development of metastatic disease was used as a control during qPCR experiments.

### 2.9. ABHD6 Immunohistochemistry

Immunohistochemistry was performed with an automated immunohistochemistry staining system (Ventana BenchMark ULTRA, Ventana Medical Systems, Tucson, AZ, USA) using the alkaline phosphatase method and a red chromogen. In brief, following deparaffinization and heat-induced antigen retrieval for 64 min, the tissue sections were incubated with a rabbit polyclonal antibody raised against synthetic peptide of human ABHD6 (1:100, MyBioSource, San Diego, CA, USA) for 1 h at 37 °C, followed by red detection and counterstain with hematoxylin II and bluing reagent according to the manufactures instructions (Ventana). Kidney, tonsil, and the retinal pigment epithelium were used as positive controls for *ABHD6* expression. An ophthalmic pathologist independently evaluated the histopathological characterization of the tissue sections and the immunohistochemistry staining. For every section, an immunoreactive score (IRS) was determined. We first determined the intensity of the cytoplasmatic staining (absent, mild, moderate, and intense, scored as 0, 1, 2, or 3, respectively). Next, the percentage of stained cells that showed the predominant intensity, was determined; no positive cells were scored as 0% and less than 10%, 10% to 50%, 51% to 80%, and more than 80% were scored as 1, 2, 3, or 4, respectively. Then, IRS was calculated by multiplying the score for percentage of stained cells with the score for the intensity of the staining [41].

### 2.10. Statistical Analysis and Code Availability

Analysis was performed using the R statistical platform language (v3.6.3). All used custom R code can be freely requested by contacting the authors.

## 3. Results

### 3.1. Establishing a Uniform Clinical Dataset of UM from Various Studies

Updating the ROMS database [11] resulted in seven additional *SF3B1*^mut^ samples. One UM sample classified as *SF3B1*^mut^ UM in the Yavuzyigitoglu et al. set was excluded due to melanoma originating from Nevus of Ota. We hypothesized clinical or genetic variables could be indicative for either early-onset metastatic disease *SF3B1*^mut^ UM (PFS < 60 months) or *SF3B1*^mut^ UM patients with late-onset metastatic disease (PFS ≥ 60 months). Therefore, we acquired 761 UM from a total of 11 large-scale UM studies (Supplementary Figure S1) and generated a uniform dataset that comprised 146 *SF3B1*^mut^ UM (Supplementary Table S1).

### 3.2. Overview of the Clinical Parameters of the ROMS Cohort

The ROMS cohort (*n* = 48) consisted of 21 males and 27 females with mean age at diagnosis of 56.7 years (±16.8 standard deviation (SD)). The mean largest tumor diameter (LTD) was 13.6 mm (±3 SD), and mean tumor thickness was 6.8 mm (±2.6 SD). Age at diagnosis did not differ between the ROMS and non-ROMS cohorts (*p* = 0.836). However, LTD, tumor thickness, inflammation, and necrosis were significantly different (all *p*-values smaller than 0.05) between the ROMS and non-ROMS UM patients (Supplementary Table S2). Finally, mean PFS was 93.5 months (±57.2 SD) in the ROMS cohort, which was significantly longer (*p* < 0.001) compared to the mean PFS in the non-ROMS cohort of 45.4 months (±33.6 SD). Demographic and genetic variables per dataset are shown in Supplementary Table S1, and these variables stratified for ROMS vs. non-ROMS data are shown in Supplementary Table S2A.

### 3.3. Stratification of All SF3B1^mut^ UM

**Figure 2:**
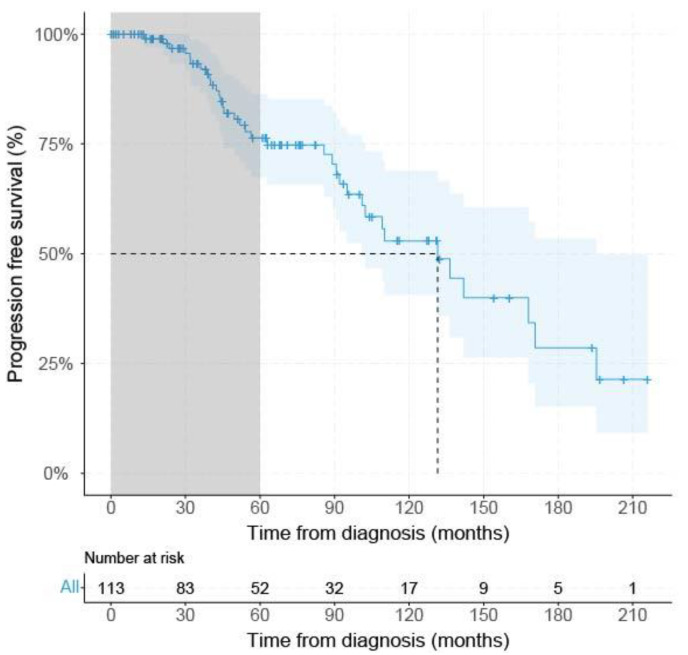
Kaplan–Meier survival curve of all *SF3B1*^mut^ UM. Grey area indicates PFS < 60 months. Dashed gray line indicates median progression free survival percentage with corresponding time from either diagnosis or treatment, dependent on the description in the original papers. Blue line shows progression-free survival with a confidence interval of 95% indicated by the blue area and censored data indicated by vertical bar.

From the 146 acquired UM, survival data were available for 113 patients, 19 patients had PFS < 60 months (with metastatic disease), and 52 UM patients had PFS ≥ 60 months. Only the mean largest tumor diameter was significantly (*p* < 0.001) larger (17.7 mm (±2.8 SD) in the PFS < 60 group compared to the PFS ≥ 60 months group (mean: 14.7 mm (±3.7 SD) (Table 1). The median PFS of all *SF3B1*^mut^ UM at risk (*n* = 113) was 131.5 months (95% CI: 101.0–195.4) using Kaplan–Meier survival analysis (Figure 2).

### 3.4. Metastatic Location of SF3B1^mut^ UM

Within the ROMS cohort, 15 UM patients developed metastatic disease mostly located in the liver (*n* = 8). However, two patients developed both liver and lung metastasis. Two patients showed liver and ossal metastases, and one other patient was diagnosed with only ossal metastasis. One patient had liver and pancreatic metastases. Finally, there was one patient that developed lung, liver, kidney, and subcutaneous metastases (Supplementary Table S2A). In all other datasets, the reported metastatic location in *SF3B1*^mut^ UM was the liver (Supplementary Table S1), without specifying other metastatic locations.

### 3.5. Differential Analysis of Canonical Transcripts Reveals SF3B1^mut^-Exclusive Transcripts

**Figure 3:**
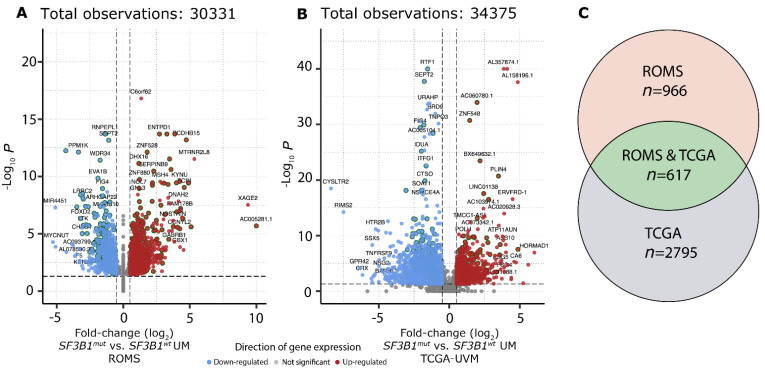
Differential gene expression in *SF3B1*^mut^ UM. (**A**) Volcano plot of the differential expression analysis between *SF3B1*^mut^ (*n* = 12) and *SF3B1*^wt^ (*n* = 14) UM within the ROMS cohort. Genes significantly down-regulated (blue) and up-regulated (red) in *SF3B1*^mut^ UM are shown. Gene names for the top 50 genes (based on descending -log_10_ q-value) and top 20 (based on |log_2_ fold change|) for both directions are shown. Genes that were found to be differentially expressed in both cohorts (a and b; *n* = 617) are highlighted by a dark green outer circle. The *x*-axis displays the log_2_ fold-change and *y*-axis displays the adjusted *p*-value (q) on a -log_10_ scale. The total amount of tested genes is shown on top. (**B**) Same as a), except for the differential expression analysis between *SF3B1*^mut^ (*n* = 15) and *SF3B1*^wt^ (*n* = 61) UM within the TCGA-UVM cohort. (**C**) Venn diagram displays differential gene expression results for ROMS, ROMS and TCGA, and TCGA cohorts.

To investigate which canonical transcripts are differentially expressed between *SF3B1*^wt^ and *SF3B1*^mut^ in UM, we performed independent differential gene expression analysis on the ROMS (Figure 3A) and TCGA-UVM (Figure 3B) cohorts, respectively, which resulted in 617 gene candidates with a *p*-adjusted value < 0.05 in both the ROMS and TCGA-UVM cohort independently (Figure 3C). Of these 617 differentially expressed genes, 37 are known cancer genes, of which 21 were downregulated and 16 were upregulated (Supplementary Table S3). Gene-set enrichment analysis (GSEA) revealed upregulation of the spliceosome machinery and downregulation of inflammatory response in both cohorts. Moreover, the downregulation of chemokine signaling, T-cell receptor signaling, and natural killer cell-mediated cytotoxicity pathways within *SF3B1*^mut^ UM suggests less inflammatory activity in *SF3B1*^mut^ UM compared to *SF3B1*^wt^ UM (Supplementary Figure S4, Supplementary Table S4).

### 3.6. SF3B1^mut^ UM Can Be Stratified Using Differentially Expressed Canonical Transcripts in PFS < 60 and PFS ≥ 60 Months

**Figure 4:**
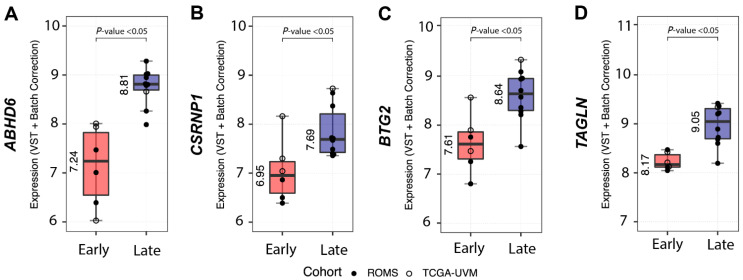
Differential gene expression between *SF3B1*^mut^ with short PFS < 60 (Early-onset) vs. *SF3B1*^mut^ with a long PFS ≥ 60 months (Late-onset). Overview of the most differentially expressed genes between *SF3B1*^mut^ with an PFS < 60 from the combined (ROMS and TCGA-UVM) cohort. ROMS samples are depicted with closed circles, and TCGA-UVM are depicted with open circles. The boxplots show the variance stabilizing transformation (VST-transformed and batch corrected) expression per PFS category and metastatic status for (**A**) *ABHD6*, for (**B**) *CSRNP1*, for (**C**) *BT2G,* and for (**D**) *TAGLN*. All results show *p*-adjusted value < 0.05.

Analysis of differentially expressed full-length transcripts within the *SF3B1*^mut^ UM group in ROMS and TCGA datasets resulted in 14 gene candidates of which four (*ABHD6*, *CSRNP1*, *BTG2* and *TAGLN*) showed decreased expression in the PFS <60 UM group and increased expression in the PFS ≥ 60 months *SF3B1*^mut^ UM group (Figure 4, Supplementary Table S5).

**Figure 5:**
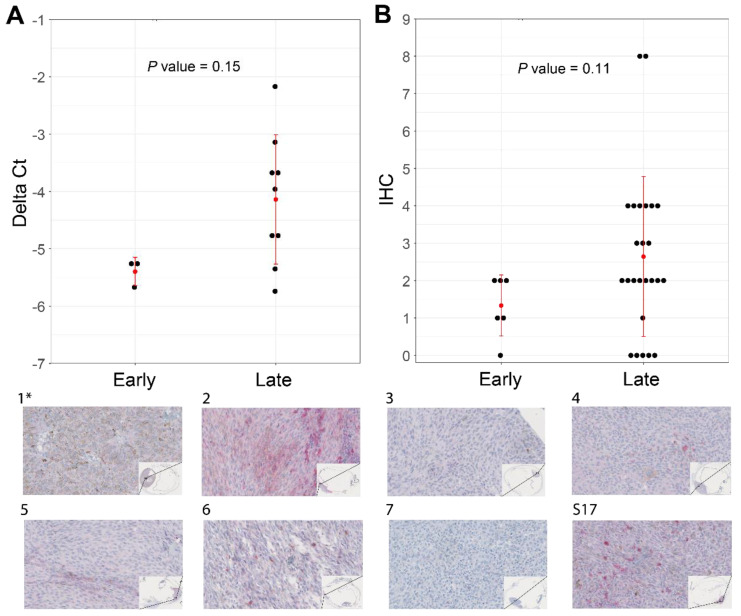
Increased levels of ABHD6 can be associated with *SF3B1*^mut^ UM with late-onset metastatic disease (PFS ≥ 60 months). (**A**) RT-qPCR performed in triplicates using *CHMP2A* expression as a normalizer of 10 primary UM (see Supplementary Figure S5). (**B**) ABHD6 IHC on all *SF3B1*^mut^ UM samples that were available and stratified for early- and late-onset metastatic disease. Red error bars represent standard deviation and red dot represents mean. Wilcoxon rank sum test was used to evaluate statistical difference of delta Ct values and IHC scores shown in scatterplots in panel A and B. *p*-value < 0.05 was considered statistically significant. (1*–7 and S17) ABHD6 IHC staining of eight primary UM samples, which are a selection of samples in panel A and B. The corresponding delta Ct values and IHC values with regard to histology are represented in Supplementary Figure S5. (40× magnification). (*) indicates the control sample with a PFS < 60 months.

Our attempts to validate the differential expression of *ABHD6* and *CSRNP1* transcripts with IHC and RT-qPCR was hampered due to the scarcity of N2-stored tumor tissue. Nevertheless, we could collect material for IHC and RT-qPCR from ten *SF3B1*^mut^ UM samples: one sample had a PFS < 60 months, and nine belonged to the PFS ≥ 60 months group. The expression of *ABHD6* was, in general, higher in all validation samples compared to a short PFS reference sample, except for one RT-qPCR sample and for two ABHD6 IHC scores (Figure 5A). Mean delta Ct value was −5.38 (±0.25 SD) in the PFS < 60 months patient sample compared to the mean delta Ct value of −4.14 (±1.13 SD) in the PFS ≥ 60 months group, which was not significantly different between the two groups (*p* = 0.15) (Figure 5A), and the mean IHC value for the PFS < 60 months group was 1.34 (±0.82 SD) compared to the mean IHC of 2.64 (±2.14 SD), which was also not statistically different between the groups (*p* = 0.11) (Figure 5B). The RT-qPCR expression of *CSRNP1* could not validate the results from our in silico differential gene expression analysis.

### 3.7. Differential Analysis of Aberrant-Splicing Reveals SF3B1^mut^-Exclusive Transcripts

Using our custom DEXSeq pipeline, we next investigated the value of differential (novel) exon usage to discriminate between *SF3B1*^mut^ and *SF3B1*^wt^ UM within the ROMS cohort, whilst again using the UVM-TCGA with the same design as validation cohort. Comparing *SF3B1*^mut^ vs. *SF3B1*^wt^ samples within the ROMS revealed 2107 differentially expressed exonic regions from 1353 distinct genes, of which 397 exonic regions from 257 distinct genes were also found differentially expressed within the TCGA-UVM (Supplementary Figure S6A–C, Supplementary Table S6). Of these 397 shared differentially expressed exonic regions, we could detect 78 novel acceptor (63 distinct genes) and 19 novel donor (14 distinct genes) splicing aberrations not present within the canonical transcript annotations (GENCODE v30). Amongst these 77 genes containing novel acceptor and donor splicing aberrations, we found known onco- and tumor-suppressor genes such as *CDK2*, *BRD9*, *NACA*, *ZNF638*, *PPP2R5A*, *NONO*, *STIP1*, and *SMARCD2*, as well as 21 onco- and tumor-suppressor genes with other forms of differential exon usage (Supplementary Table S6). Using the exon-overlapping read counts of these 77 genes and performing unsupervised clustering (Euclidean distances and Ward.D2 method) on all samples within the ROMS cohort revealed clear separations of *SF3B1*^mut^ UM vs. *SF3B1*^wildtype^ (Supplementary Figure S7).

### 3.8. SF3B1^mut^ UM Can Be Stratified Using Differentially Expressed Aberrant Transcripts in PFS < 60 and PFS ≥ 60 Months

In line with the discriminatory value of using differential exon usage to distinguish *SF3B1*^mut^ and *SF3B1*^wt^ UM, we hypothesized whether similar patterns could be indicative of early- or late-onset of metastatic disease in *SF3B1*^mut^ UM (PFS < 60 months vs. PFS ≥ 60 months) within the ROMS cohort.

**Figure 6:**
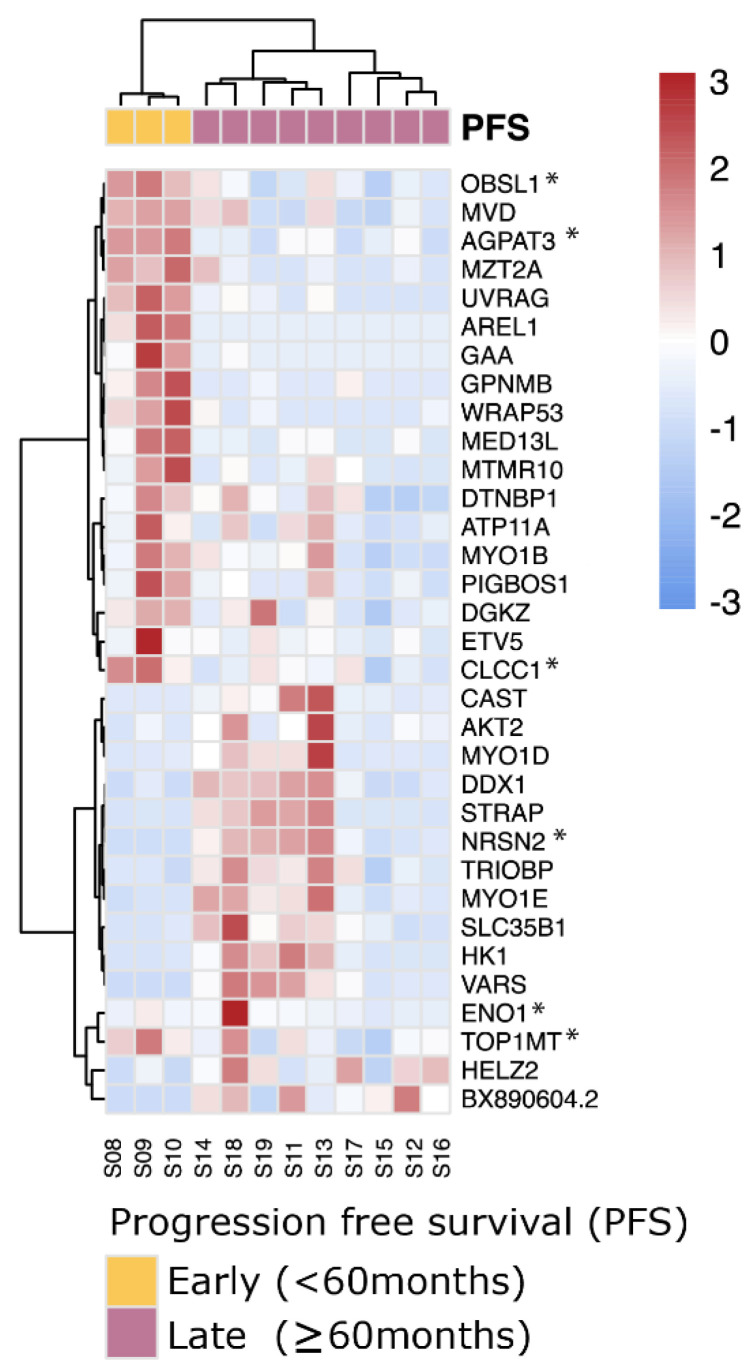
*SF3B1*^mut^ UM is characterized by aberrant splicing events. Unsupervised clustering (Euclidean distances and Ward.D2 method) on the differential exon usages between *SF3B1*^mut^ samples in the ROMS cohort with PFS < 60 months and PFS ≥ 60 months. Values are mean-centered read counts represented with a z score of differentially expressed splicing events in genes (blue, low expression; red, high expression). Top bars represent PFS-status (yellow; PFS < 60 months and purple; PFS ≥ 60 months). Asterisk (*) indicates novel splicing aberrations that are either donors or acceptors.

Using the same strategy as before, albeit without the ability of using the TCGA-UVM cohort as validation because of the lack of follow up data, we could detect 34 differential genes with aberrant exon usages in 33 distinct genes between PFS < 60 months and PFS ≥ 60 months *SF3B1*^mut^ samples (Figure 6). Of these 34 events, 6 were involved with novel acceptor and donor aberrations not present within GENCODE v30 (*ENO1*, *OBSL1*, *NRSN2*, *AGPAT3*, *TOP1MT* and *CLCC1*), and in total, four genes were known onco- and tumor suppressor genes (*ETV5*, *CLCC1*, *AKT2* and *WRAP53*). These splicing events supplement the full-length transcript markers shown previously to differentiate between early- and late-onset metastatic disease of *SF3B1*^mut^ UM (Supplementary Table S7).

## 4. Discussion

In the current study, we describe the largest number of *SF3B1*^mut^ UM (*n* = 146) in the literature and report on the prognostic value of clinical and genetic variables, whilst distinguishing between early (PFS < 60 months) and late (PFS ≥ 60 months) metastatic onset *SF3B1*^mut^ UM. This could provide us with tools to suggest a more stringent treatment protocol or inclusion in specific clinical trials. The question remains if these potentially more stringent screening programs will result in the prolonged overall survival of *SF3B1*^mut^ UM patients. However, after validation of our results in external cohorts, low expression of ABHD6 could potentially be used as a biomarker to detect and select high-risk *SF3B1*^mut^ UM.

A high expression of *ABHD6* is indicative of a long progression-free survival (PFS ≥ 60 months), whereas a low expression is indicative of early-onset metastatic disease (PFS < 60 months). As described in previous reports, UM with somatic alterations within the spliceosome have a distinct CNV profile and are characterized by aberrant transcripts due to a mutated allele of *SF3B1*. Considering survival, we have shown that, in particular, *SF3B1*^mut^ UM gives rise to late-onset metastases [4]. Nevertheless, the *SF3B1*^mut^ UM patients may also show a less favorable prognosis with early-onset metastatic disease. Although, the majority of the samples with a PFS > 60 months originated from our cohort (*n* = 9), these UM patients were also present within the Alsafadi et al. (*n* = 1), Rodrigues et al. (*n* = 3), Shain et al. (*n* = 1), and Harbour et al. (*n* = 2) UM cohorts. Even though this constitutes only a limited number of UM patients within each external cohort, it does illustrate that late-onset metastatic disease occurs in *SF3B1*^mut^ UM, but only when study follow-up is sufficient. From the clinical parameters, only LTD was significantly larger in the early-onset metastatic UM group compared to the late-onset metastatic UM, which corroborates with earlier study findings, where tumor size was shown to be an independent predictor of UM aggressiveness [17,42]. All included samples in our analyses are mainly derived from larger tumors (Table 1), yet smaller tumors are also represented in our cohort. Consequently, tumor size difference between studies may vary due to selection bias, whereas the discrepancy in necrosis and inflammation classification could be based on non-uniform definition of these variables. When comparing the ROMS *SF3B1*^mut^ to the other studies for metastases, we did not observe significant differences between clinical and pathological parameters. Interestingly, the ROMS patients have a longer follow-up in contrast to the other studies, possibly explaining difference in median PFS. Regarding metastatic location, there were no associations between extrahepatic metastatic locations and high risk or low risk *SF3B1*^mut^ UM, most likely due to a restricted number of metastatic samples.

### 4.1. Unbiased Detection of Pathogenic SF3B1^mut^ UM

In contrast to *BAP1* mutations, *SF3B1* mutations are change-of-function mutations rather than loss-of-function mutations, with the R625 alteration being the predominant hotspot in UM. However, mutations at other locations encoding K666 or K700 also occur within smaller proportions of UM. At present, it is not clear whether mutations outside the common SF3B1 hotspots are merely passenger mutations, or if these contribute to UM pathogenesis warranting the need for an unbiased diagnostic approach capable of detecting compromised SF3B1 function. One such method could be based on a straightforward PCR primer assay, designed to detect *SF3B1*^mut^-specific aberrant transcripts indicative of the transcriptomic hallmark of *SF3B1*^mut^ UM. This could broaden our understanding and inventory of *SF3B1*^mut^ UM.

Downstream effects of the *SF3B1*^mut^ allele are probably vast due to the amounts of aberrant transcripts that are formed. For example, the tumor suppressor BRD9 that is linked to melanomagenesis was recently shown to be downregulated in *SF3B1*^mut^ UM due to the incorporation of a poison exon, which leads to the non-sense mediated decay of *BRD9* [43]. Most likely, there are multiple transcripts that behave as the aforementioned tumor suppressor and are interesting to study. Therefore, differential gene expression in combination with non-sense mediated decay inhibition and queries for stop sequences outside canonical exon borders with sufficient coverage could aid in the detection of novel tumor suppressors. Interestingly, in our differential gene expression analysis, we observed that only *ABHD6* expression can be used to discriminate between early-onset and late-onset metastatic *SF3B1*^mut^ UM. ABHD6 is a lipase involved in endocannabinoid signaling and possibly has other effects in inflammation, metabolic syndromes, and insulin secretion. In contrast to earlier publications [44,45], our results show that the decreased expression of *ABHD6* is associated with high-risk *SF3B1*^mut^, which we validated using qPCR and IHC in nearly all UM cases tested. However, in order to implement this biomarker in clinical settings, more samples should be tested in another study with sufficient follow-up. For now, the results are promising, and it is possible to identify early onset metastatic *SF3B1*^mut^ UM.

A limitation of our differential expression analyses to draw convincing conclusions are the low number of samples. However, 617 differentially expressed genes were observed between *SF3B1*^mut^ and *SF3B1*^wt^ UM (concordant in both the ROMS and TCGA-UVM cohort), of which 37 are known cancer genes. In addition, GSEA revealed perturbations within spliceosome machinery and inflammatory responses. Moreover, downregulation of chemokine signaling, T-cell receptor signaling, and natural killer cell-mediated cytotoxicity pathways within *SF3B1*^mut^ UM could indicate a decreased inflammatory function, reflecting potential immunological differences [12]. Furthermore, utilizing the transcriptomic hallmark of dysregulated splicing due to *SF3B1* mutations, we could identify 2107 *SF3B1*^mut^-specific or significantly perturbed splicing aberrations with donor and/or acceptor extensions or shortenings.

### 4.2. Potential Biomarkers Capable of Distinguishing between Early- and Late-Onset SF3B1^mut^ UM

Using differential expression analysis on canonical transcripts and detection of *SF3B1*^mut^-specific alternatively spliced transcripts between early- and late-onset *SF3B1*^mut,^ we identified putative biomarkers between these groups of patients. We detected four canonical transcripts (*ABHD6*, *CSRNP1*, *BTG2*, and *TAGLN*) with significant up-regulated expression within the late-onset UM compared to early-onset UM. We set out to validate the expression of *ABHD6* using IHC and rt-qPCR on secondary UM samples. The expression of *ABHD6* varies between cancer types and is increased in Ewing sarcoma, prostate cancer, Burkitt lymphoma, and leukemia [46,47]. Moreover, *ABHD6* expression in early metastatic UM was significantly downregulated compared to primary non-metastatic uveal melanoma, which corroborates our study findings within *SF3B1*^mut^ UM [48]. Preferably, more PFS < 60 months *SF3B1*^mut^ UM cases should be included to draw a convincing conclusion that ABHD6 expression is a prognostic biomarker of PFS in *SF3B1*^mut^ UM. Due to a lack of IHC staining in samples 3 and 7, we cannot fully conclude ABHD6 IHC is a reliable test to differentiate between early- and late-onset metastasis in *SF3B1*^mut^ UM, and unfortunately, these results do not corroborate the more reliable qPCR results. An explanation for discrepancy between qPCR and IHC staining in sample 3 and 7 is the fact that the RNA was isolated from a different (N_2_ stored) part of the tumor, and we cannot exclude some heterogeneity in the tumor specimen. However, pooled data of qPCR results and IHC signaling do support our conclusion that high ABHD6 expression is characteristic for late-onset metastatic disease in *SF3B1*^mut^ UM. The ABHD6 gene maps to chromosome 3 and the possible involvement of (partial) chromosome 3 deletions could play a role in the ABHD6 deficiency and shorter progression-free survival. However, the log_2_fold changes are much more than expected from simply losing a copy of the chromosome, nor did we observe a significant change of expression in other chromosome 3 genes such as *BAP1*. We also detected 34 significantly perturbed splicing aberrations with donor and/or acceptor extensions or shortenings between early- and late-onset metastatic *SF3B1*^mut^ UM. These findings warrant further research in utilizing such biomarkers to develop classification schemes to distinguish *SF3B1*^mut^ UM patients with potential early- and late-metastatic onset.

## 5. Conclusions

This study describes clinical and genetic characteristics of the largest aggregated cohort of *SF3B1* mutated UM in literature (*n* = 146). Patients with *SF3B1*-mutated UM show early- and late-onset metastatic disease (defined as before or after a follow-up time of 60 months), for which, currently, no biomarkers exist, and for which we identified several promising candidates with potential distinguishing characteristics when implemented within an expression-based classifier. In addition, the largest tumor diameter (LTD) was found to be increased in the early-onset UM. Our results provide more accurate prognostication and targets for future functional studies in an effort to elucidate pathogenesis and clinical stratification of *SF3B1*-mutated UM.

## Figures and Tables

**Table 1:** All *SF3B1*-mutated UM stratified for PFS < 60 months and PFS ≥ 60 months with description of clinical variables. Age at diagnosis, gender, ciliary body involvement, epithelioid cells present, extraocular extensions, closed extracellular matrix patterns, tumor thickness, T class in TNM category, inflammation, necrosis, *GNAQ* and *GNA11* status, and *SF3B1* amino acid mutation did not significantly differ between PFS < 60 months and PFS ≥ 60 months groups (all *p* > 0.05). Student’s *t*-test was used for continuous variables and Fisher’s exact test (indicated with *) was used for categorical variables. For overview of all variables, we refer to Supplementary Table S2B.

Variables	PFS < 60 Months (*n* = 19)	PFS ≥ 60 Months (*n* = 52)	Overall (*n* = 71)	PFS < 60 vs. PFS ≥ 60 Months *p*-Value
**Largest tumor diameter (millimeter)**				0.001
Mean (SD)	17.7 (±2.8)	14.7 (±3.7)	15.4 (±3.7)	
Median (Min, Max)	18 (13.9–24.0)	15 (9.0–25.0)	15 (9.0–25.0)	
Data not reported	2 (10.5%)	3 (5.8%)	5 (7.0%)	
**Metastasis (number)**				<0.001 *
Yes	19 (100%)	16 (30.8%)	35 (49.3%)	
No	0 (0%)	32 (61.5%)	32 (45.1%)	
Data not reported	0 (0%)	4 (7.7%)	4 (5.6%)	
**Metastatic location (number)**				0.510 *
Liver	11 (57.9%)	9 (17.3%)	20 (28.2%)	
Liver and other site	2 (10.5%)	4 (7.7%)	6 (8.5%)	
Ossal	1 (5.3%)	0 (0%)	1 (1.4%)	
Data not reported	5 (26.3%)	39 (75.0%)	44 (62.0%)	
**Progression free survival (months)**				<0.001
Mean (SD)	38.9 (±11.5)	109.2 (±42.2)	90.4 (±48.1)	
Median (Min, Max)	40.1 (13.3–56.4)	97.8 (61.0–215.9)	82.1 (13.3–215.9)	
**Patient status (number)**				<0.001 *
Alive	3 (15.8%)	28 (53.8%)	31 (43.7%)	
Died due to UM	14 (73.7%)	10 (19.2%)	24 (33.8%)	
Died of other cause than UM	0 (0%)	5 (9.6%)	5 (7.0%)	
Data not reported	2 (10.5%)	9 (17.3%)	11 (15.5%)	

## Data Availability

The data from The Cancer Genome Atlas (TCGA) and repository NCBI GEO Datasets are publicly accessible. The ROMS data are not publicly accessible. Our ethics committee does not allow sharing of individual patient or control genotype information in the public domain.

## Supplementary Materials

The following are available online at https://www.mdpi.com/article/10.3390/cancers14030846/s1, Figure S1: Overview of included cohorts, Figure S2: Overview of whole-transcriptome samples and general sequencing characteristics. (A) Number of included whole-transcriptome samples per cohort (ROMS and TCGA-UVM) and mutational status for *BAP1*, *SF3B1*, *EIF1AX* and *SRSF2*. (B) Boxplots of general alignment metrics, per cohort, after (re-)alignment against GRCh38 with GENCODE (v30) annotations. *Y*-axis depicts number of reads in log_10_-scale per category (*x*-axis). Per boxplot, the median value is shown (in millions; rounded). (C) Boxplots of the median transcript integrity number (medTIN), this metric provides an overview of the uniformity of the read-coverage over each GENCODE (v30) transcript. In general, medTIN scales according to the quality and sequencing technique of the sample, in which higher numbers represent samples which have a more uniform and higher read-coverage of the transcripts, Figure S3: Principal component analysis of the top 5000 most-variable genes within the analyzed whole-transcriptome cohorts. (A) The upper figure represents an overview of the row-wise variance for each gene (with a row-wide median of at least 2 reads) within the ROMS cohort, the total number of transcripts is indicated within the figure whilst the red dotted horizontal line depicts the border of the 5000 most-variable genes. The middle figure depicts the percentage of variance explained of the first 15 principal components after PCA (using the VST-transformed counts of the top 5000 most-variable genes after batch corrections) whilst lower figure displays the first two principal components. Samples are colored based on their mutational status in *BAP1*, *SF3B1* and *EIF1AX* whilst males and females are represented by closed and open dots, respectively. *SF3B1*^mut^ samples with a PFS < 60 months are highlighted by a red asterisk. (B) same as A), except for the TCGA-UVM cohort. (C) Same as (A), except for the combined ROMS and TCGA-UVM cohort for the PFS ≥ 60 and PFS < 60 months *SF3B1*^mut^ samples. Figure S4: Overview of geneset-enrichment analysis (q ≤ 0.05) between *SF3B1*^mut^ (*n* = 12) and *SF3B1*^wt^ (*n* = 14) uveal melanoma tissue within the ROMS cohort. Hallmark gene-sets are highlighted in blue. *Y*-axis depicts normalized enrichments scores (NES) in which NES < 0 means depleted in *SF3B1*^mut^ samples and vice versa. Genesets that overlap in both ROMS as TCGA cohorts are indicated with an asterisk. Figure S5: Increased levels of ABHD6 can be associated with late metastasizing *SF3B1*^mut^ UM with late-onset metastatic disease (PFS ≥ 60 months). (A) qPCR performed in triplicates using *CHMP2A* expression as a normalizer of 10 primary UM of which samples 1–7 are from an independent set whereas samples S15, S13 and S17 were used to validate our RNA-seq in silico results. Asterisk (*) indicates the control sample with a PFS <60 months and pound sign (#) indicates no tumor was present in histological sections. Missing IHC bar charts in samples 1, 3 and 7 indicate zero IHC scores, i.e., no presence of ABHD6. (B) Average results by pooling panel A with corresponding error bars that represent standard deviation. (C) ABHD6 IHC on all *SF3B1*^mut^ UM samples that were available and stratified for early- and late-onset metastatic disease. Wilcoxon rank sum test was used to evaluate statistical difference of IHC scores shown in boxplots in panel C. *p*-value < 0.05 was considered statistically significant. (1*-7 and S17): ABHD6 IHC staining of eight primary UM samples which correspond to results in panel A (40x magnification). Figure S6: (A) Volcano-plot of the differential exon usage analysis between *SF3B1*^mut^ (*n* = 12) and *SF3B1*^wt^ (*n* = 14) uveal melanoma tissue within the ROMS cohort, exonic regions significantly (q ≤ 0.05 and log_2_ fold change ≥ |0.5|) down-regulated (blue) and up-regulated (red) in *SF3B1*^mut^ uveal melanoma tissue are shown. Gene names for the top 50 genes (based on descending -log_10_ q-value) and top 20 (based on |log_2_ fold change|) for both directions are shown. Genes which were found to be differentially expressed in both cohorts are highlighted by a green circle. The *x*-axis displays the log_2_ fold-change and *y*-axis displays the adjusted *p*-value (q) shown on a -log_10_ scale. The total amount of differentially expressed exon usages is shown on top. (B) Same as (A), except for the differential expression analysis between *SF3B1*^mut^ (*n* = 15) and *SF3B1*^wt^ (*n* = 61) uveal melanoma tissue within the TCGA-UVM cohort. (C) Overlap of differential exon usages between ROMS and TCGA-UVM cohorts within the *SF3B1*^mut^ and *SF3B1*^wt^ analysis (performed per cohort). Pink color denotes the number of events (based on genomic location) found to be statistically differentially expressed within both the ROMS and TCGA-UVM cohorts. Figure S7: Unsupervised clustering of the ROMS cohort based upon novel acceptor and donor splicing-aberrations. Unsupervised clustering (Euclidean distances and Ward.D2 method) on the differential exon usages (not found within the GENCODE v30 annotation) between *SF3B1*^mut^ and *SF3B1*^wt^ samples in the ROMS cohort. Values are mean-centered read counts represented with a z score of differentially expressed splicing events (blue, low expression; red, high expression). Top bars represent PFS-status (yellow; PFS <60 months and purple; PFS ≥ 60 months) and mutation status. Table S1: overview of included samples and characteristics from all cohorts. Table S2: (A) overview of clinical characteristics of *SF3B1*^mut^ uveal melanoma from the ten publicly available datasets (non-ROMS) and the ROMS cohort. (B) overview of clinical characteristics of PFS <60 months *SF3B1*^mut^ uveal melanoma and PFS ≥ 60 months from the ten publicly available datasets. Table S3: Overview of Differential gene expression data *SF3B1*^mut^ vs. Other. Table S4: Overview of Gene Set Enrichment Analysis data *SF3B1*^mut^ vs. Other. Table S5: Overview of Differential Expression data *SF3B1*^mut^ PFS <60 vs. PFS ≥ 60 months. Table S6: Overview of DEXSeq differential expression of ROMS data *SF3B1*^mut^ vs. Other. Table S7: Overview of DEXSeq differential expression of ROMS data *SF3B1*^mut^ early vs. *SF3B1*^mut^ late.
