# Supplementary figures and images for: Identification of Early-Onset Metastasis in SF3B1 Mutated Uveal Melanoma
Source: Cancers (Basel). 2022 Feb 8;14(3):846. doi: 10.3390/cancers14030846 (PMC8834136; doi:10.3390/cancers14030846)

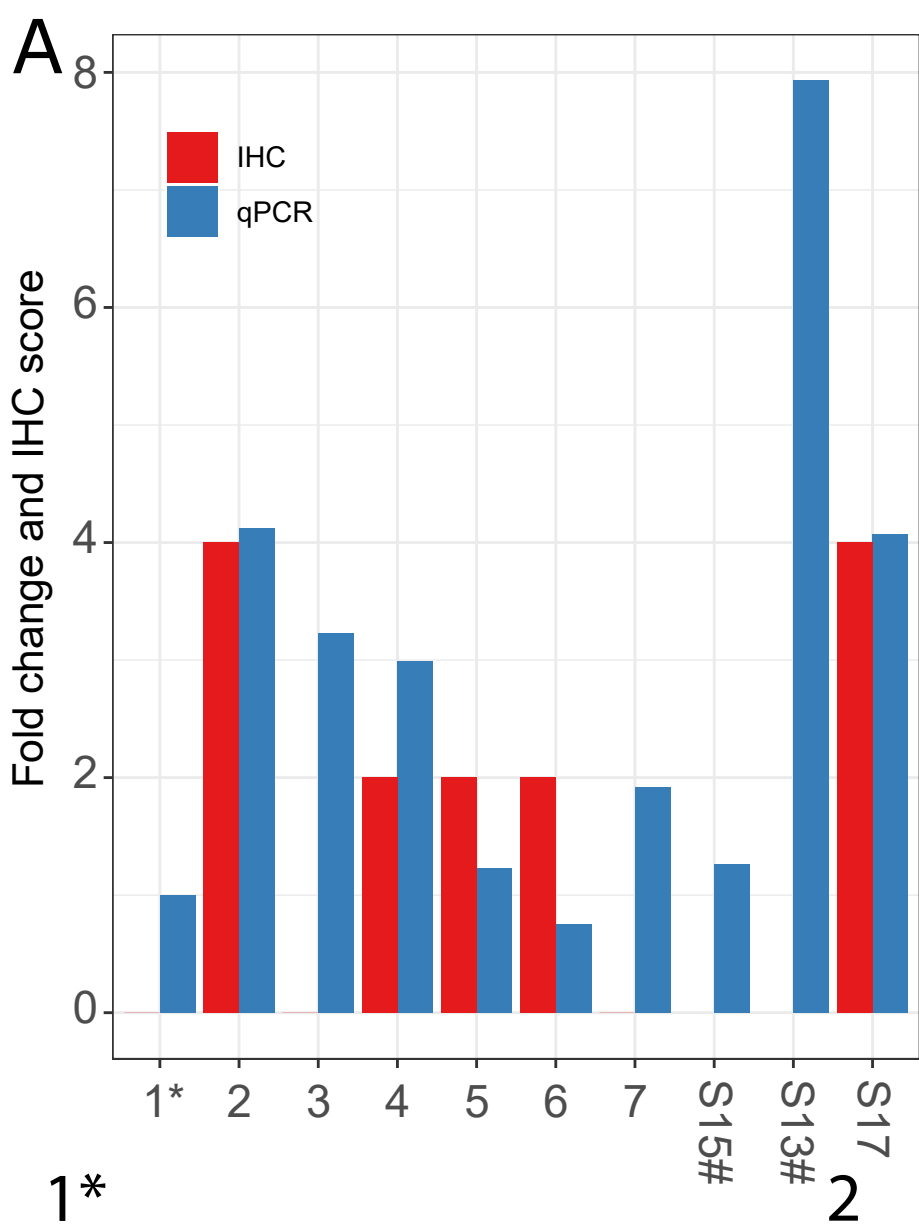

1\*

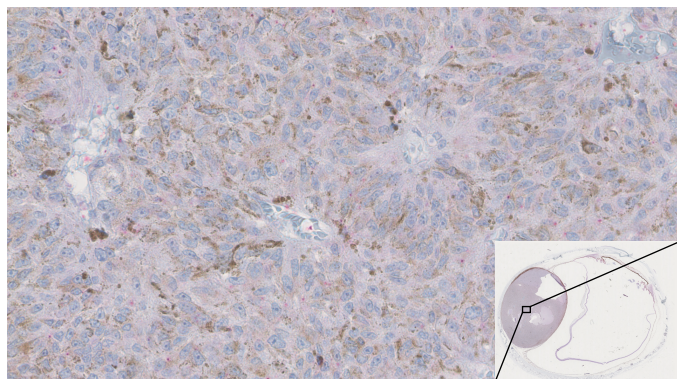

2

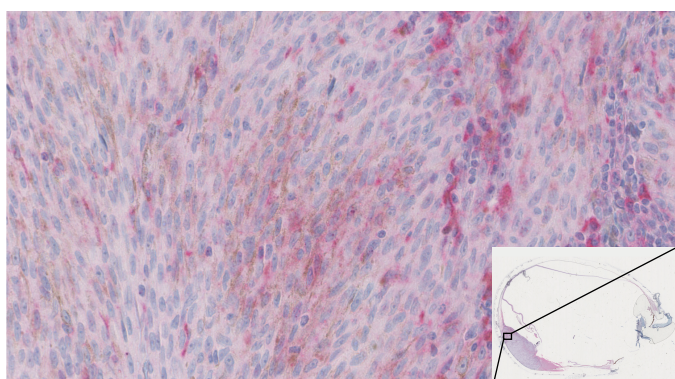

5

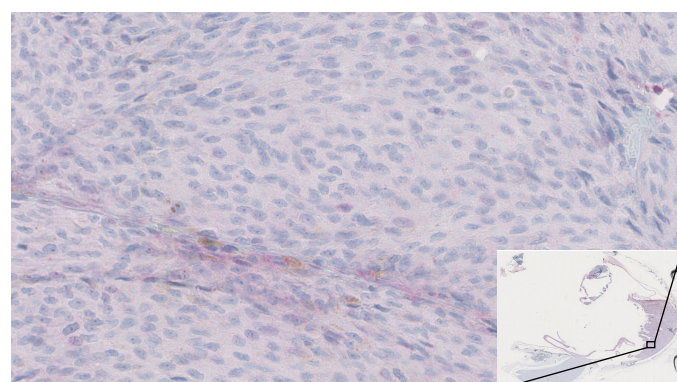

6

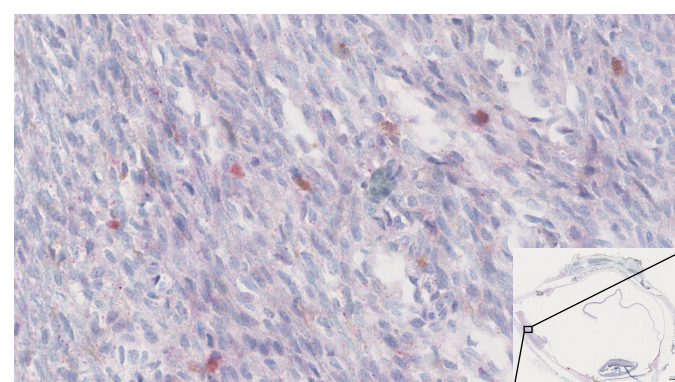

3

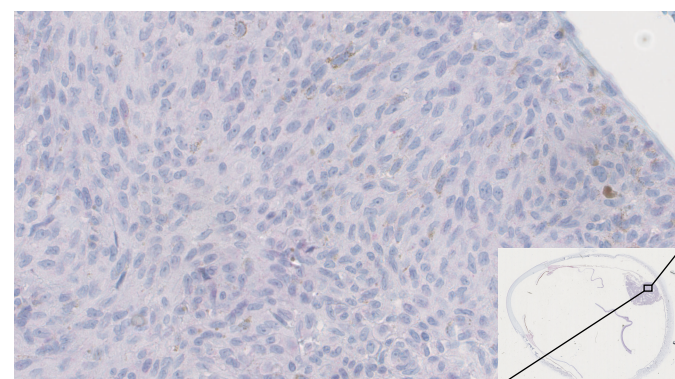

7

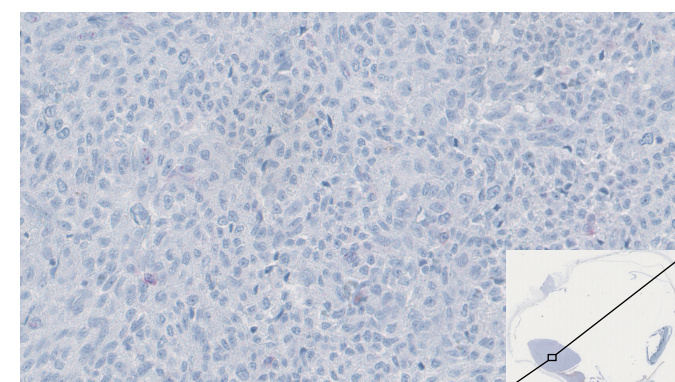

4

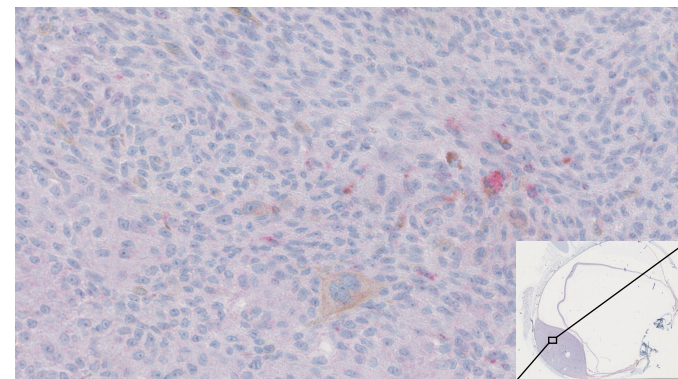

S17

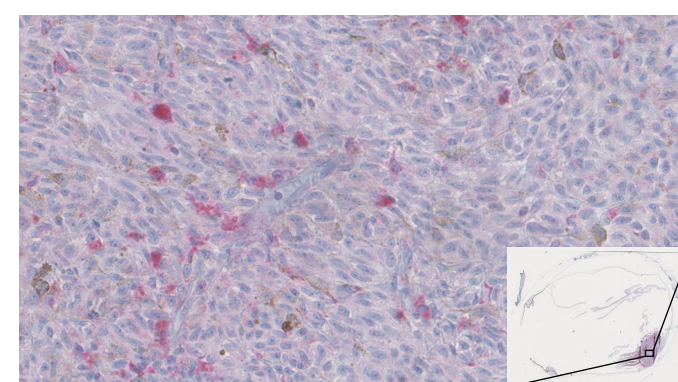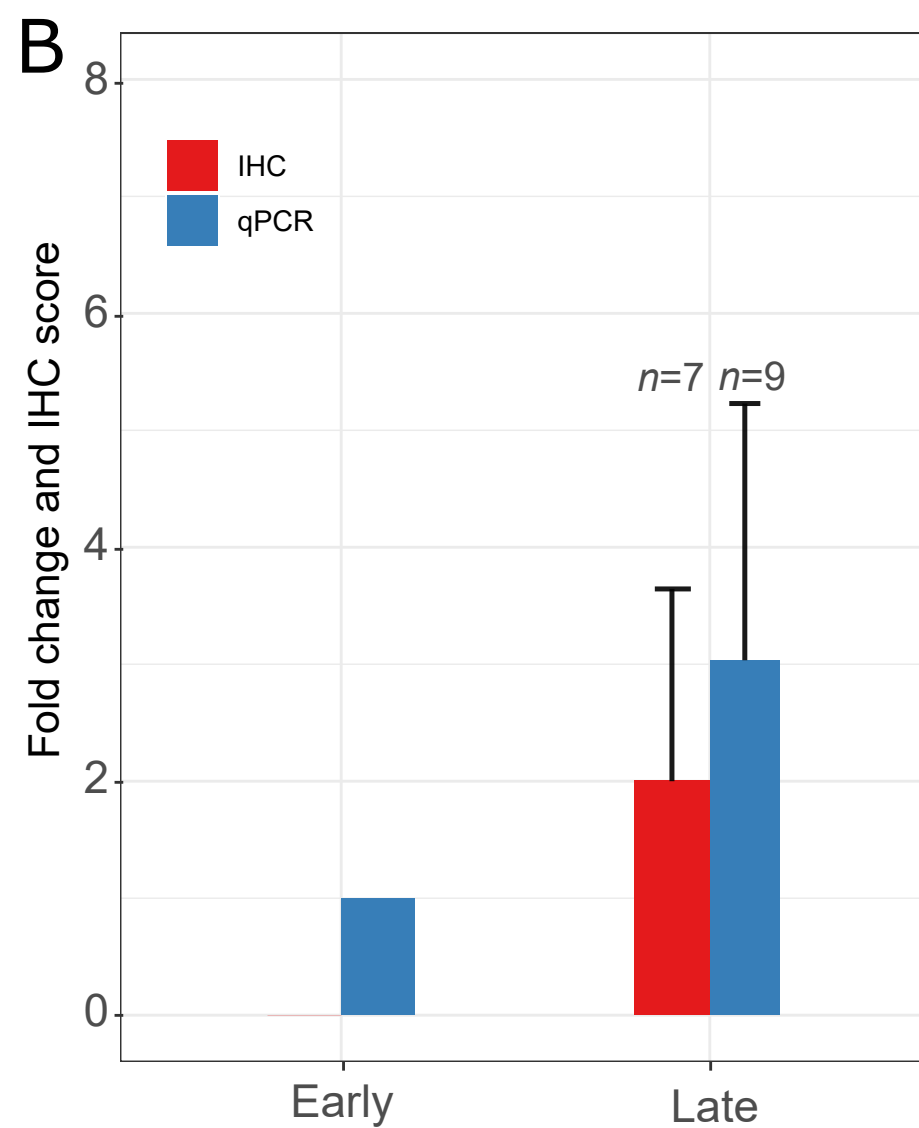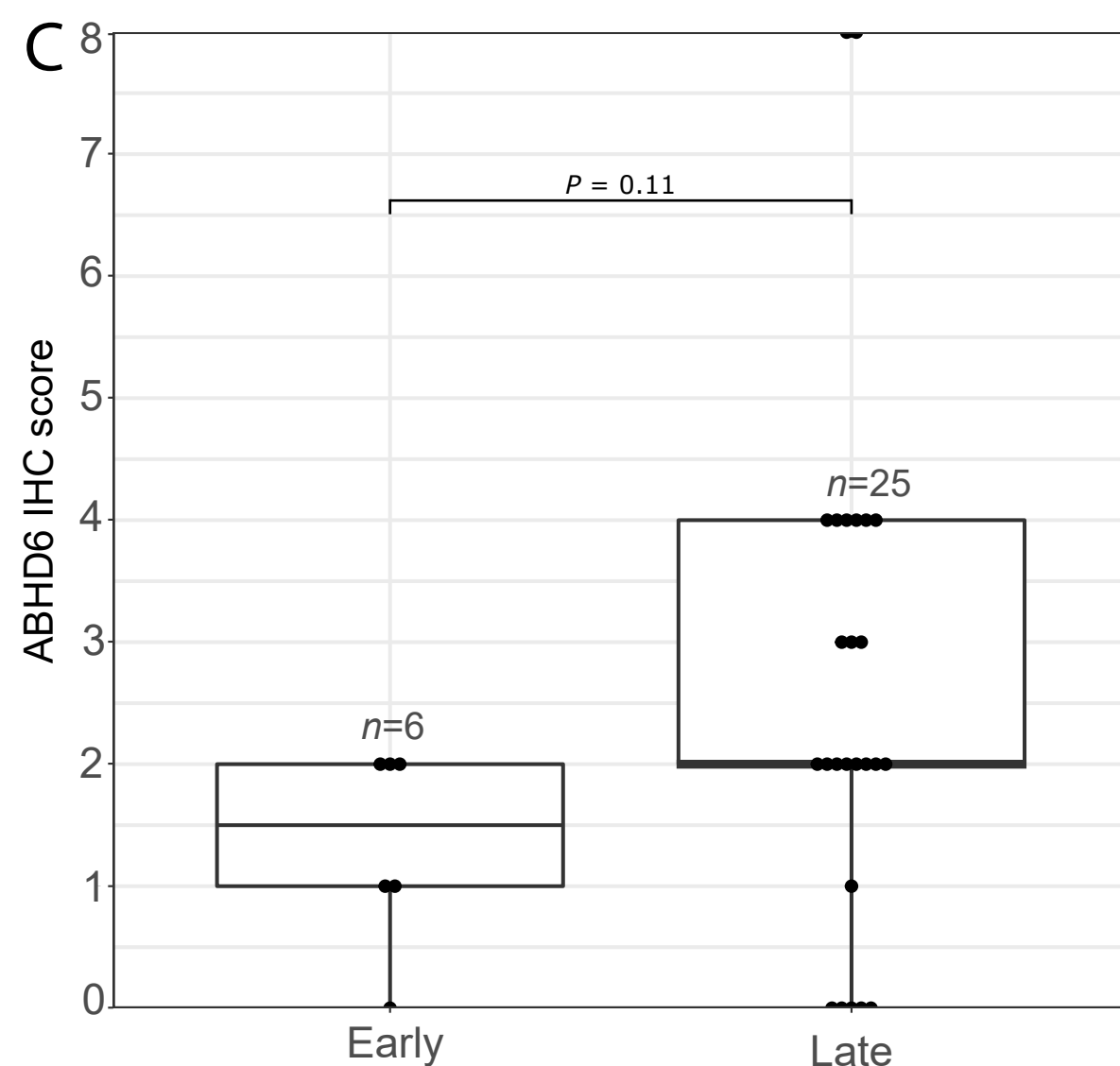

Supplement: Supplementary file 1 [file cancers-14-00846-s001.zip › SupFigS5_IHC_.pdf]

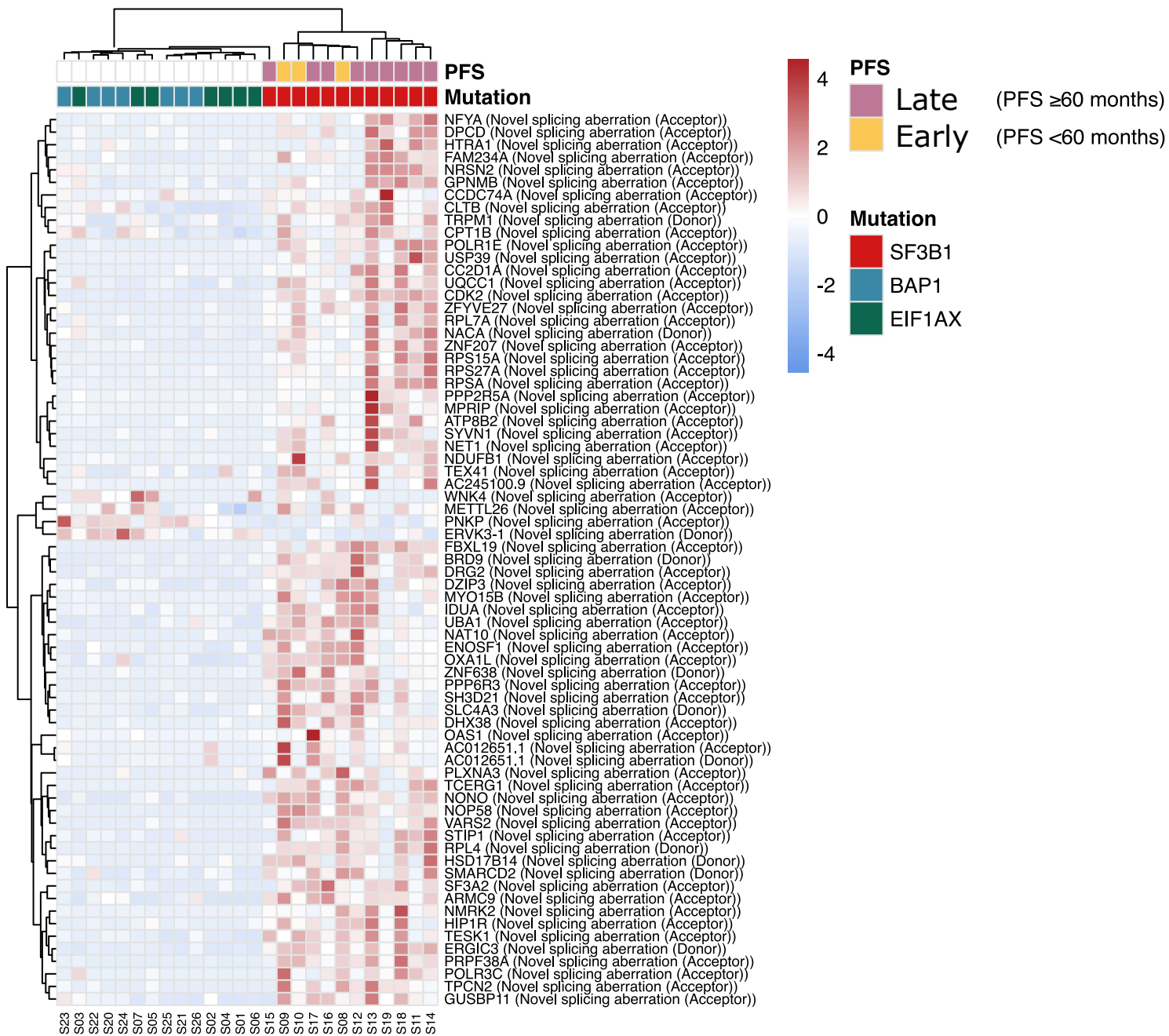

Supplement: Supplementary file 1 [file cancers-14-00846-s001.zip › SupFigS7_AbbSplicingDEXSeq.pdf]

Gene-sets  
( $q \leq 0.05$ ; Hallmark and KEGG)

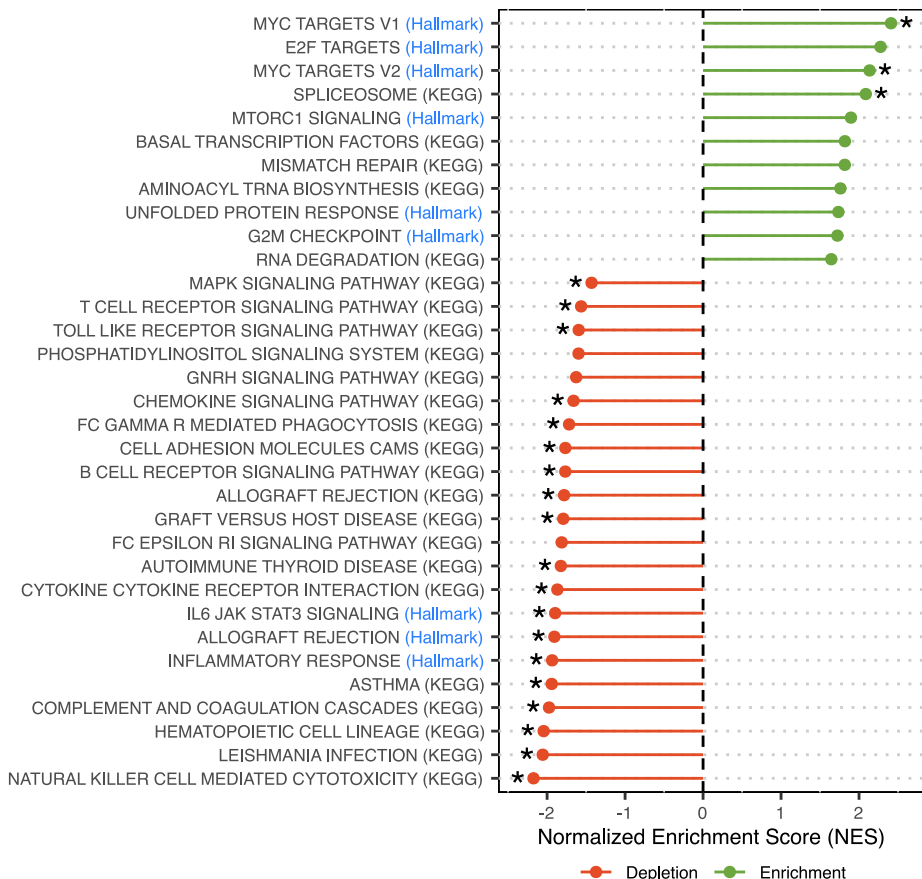

Supplement: Supplementary file 1 [file cancers-14-00846-s001.zip › SuppFigS4_gene_overlap_KEGG.pdf]

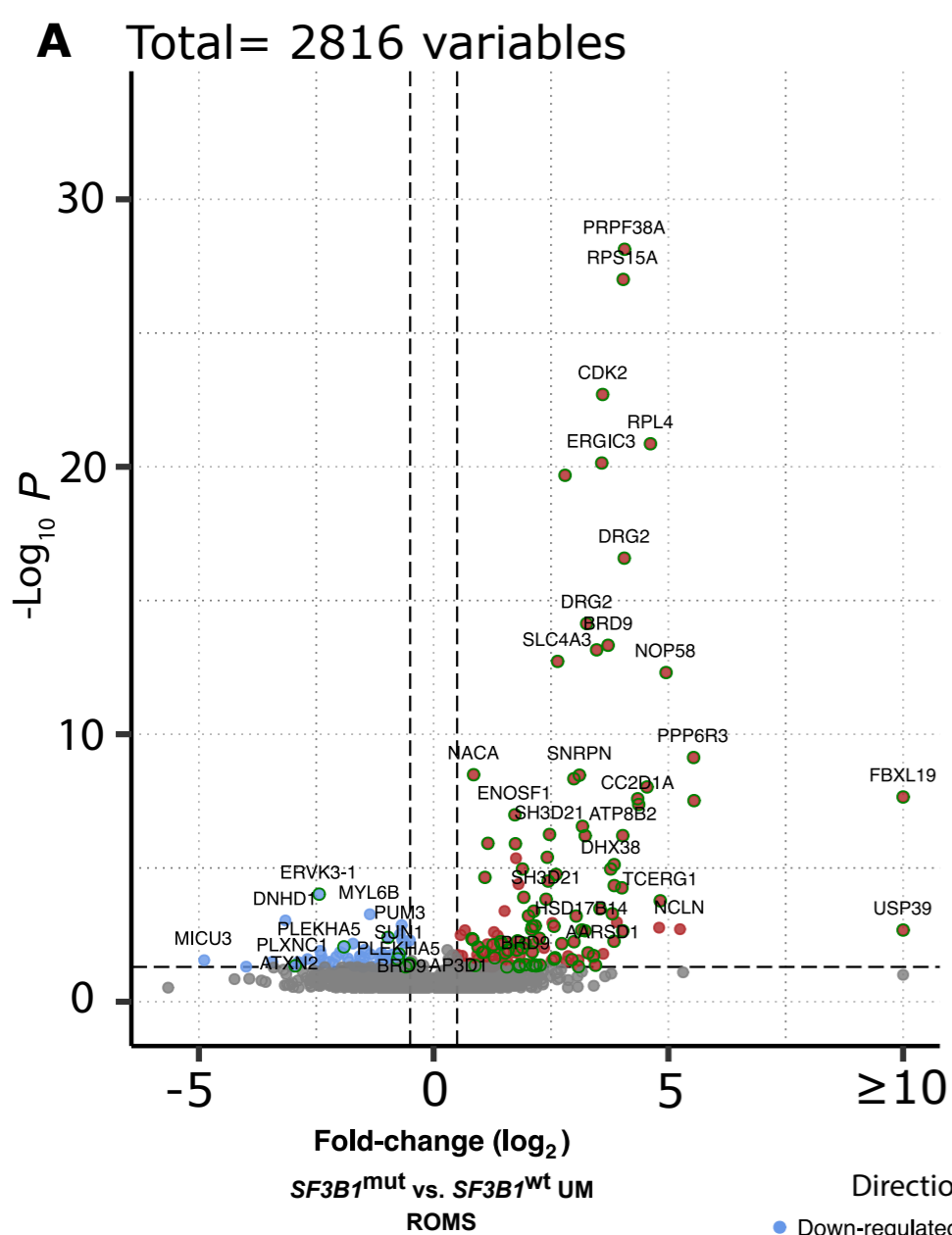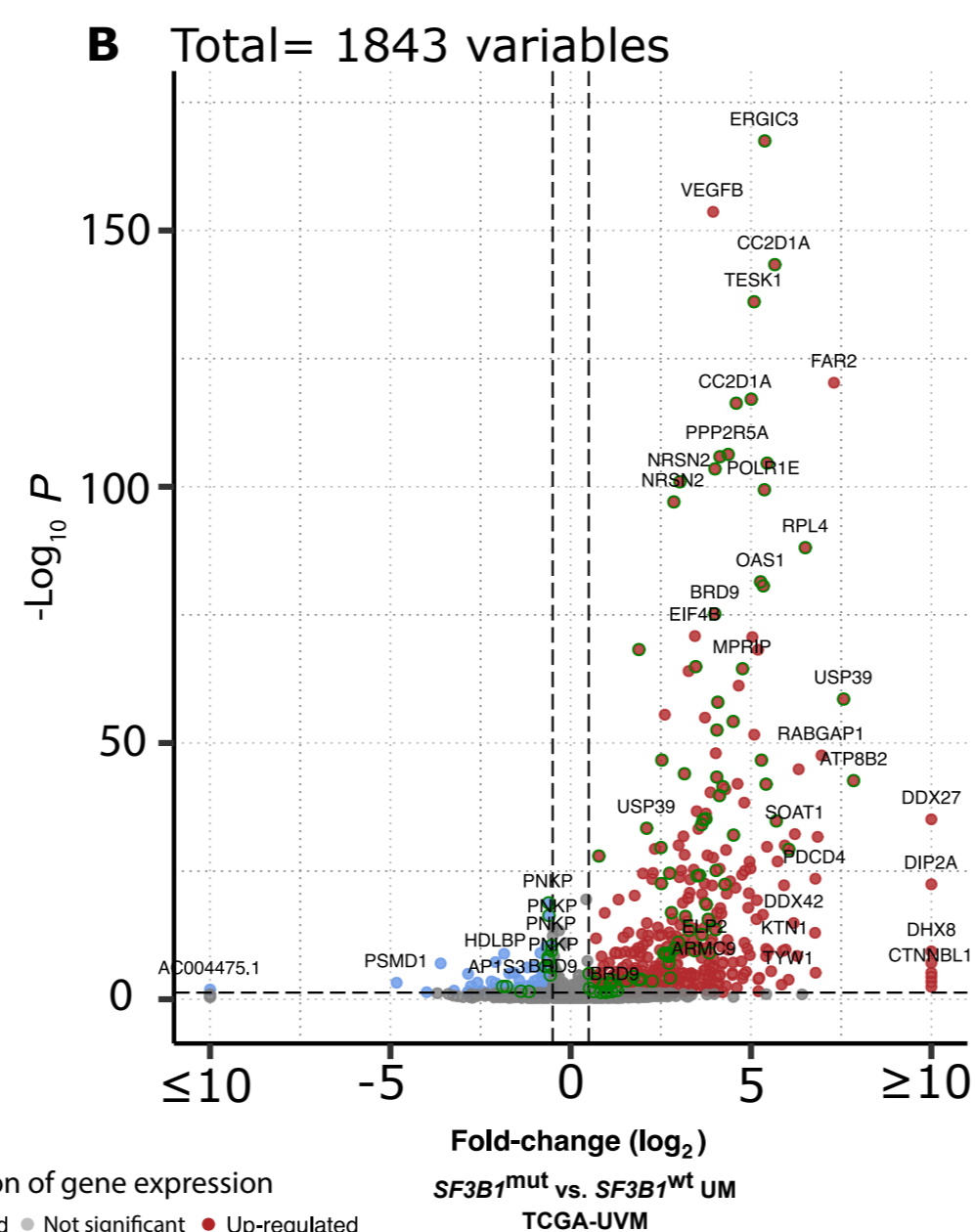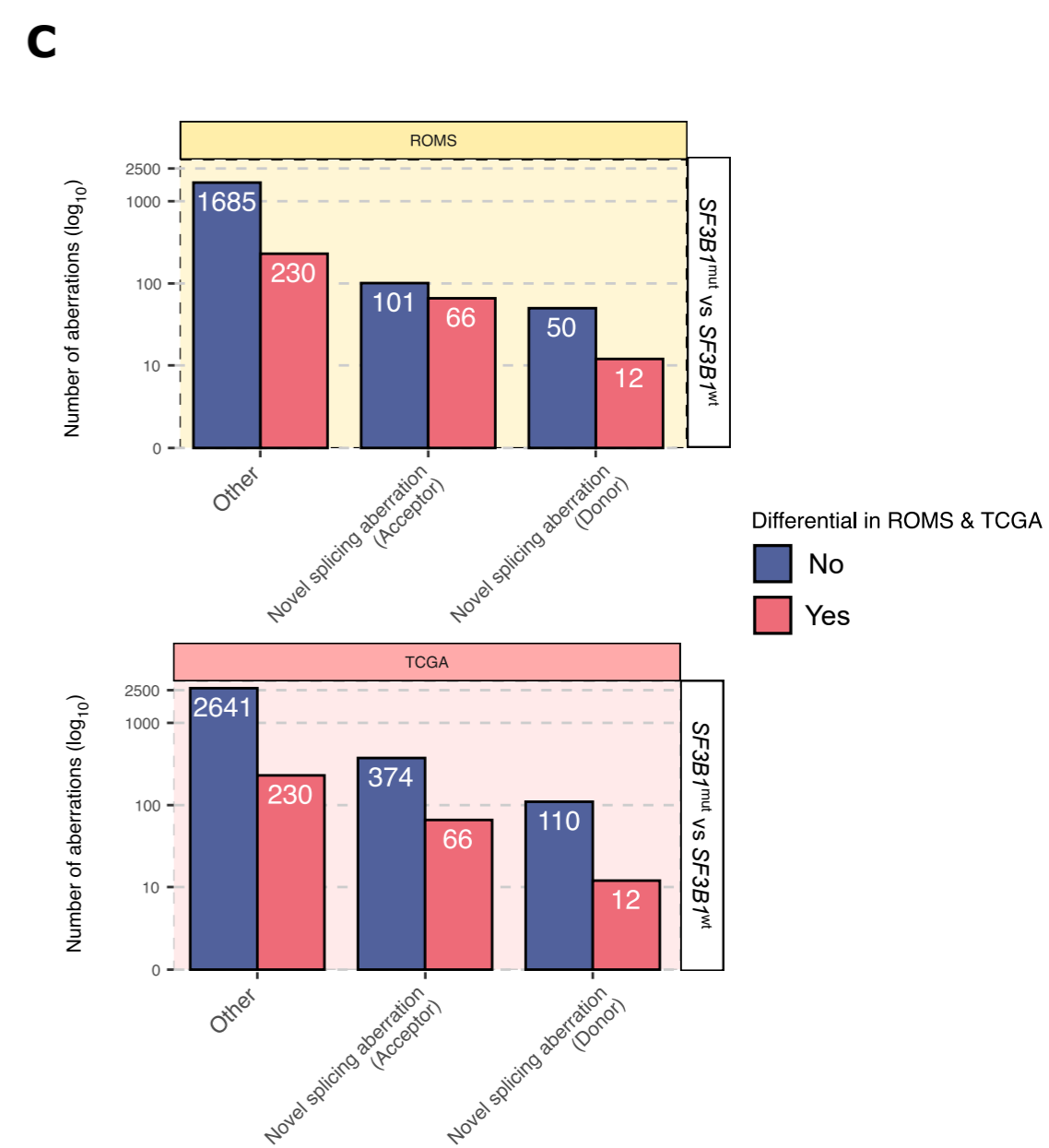

Supplement: Supplementary file 1 [file cancers-14-00846-s001.zip › SuppFigS6ABC_SplicingDefects.pdf]
